# Value of Caffeic Acid Phenethyl Ester Pretreatment in Experimental Sepsis Model in Rats

**DOI:** 10.1155/2015/810948

**Published:** 2015-04-08

**Authors:** Ozlem Alici, Havva Sahin Kavakli, Cemile Koca, Neriman Defne Altintas, Murat Aydin, Suleyman Alici

**Affiliations:** ^1^Department of Infectious Diseases and Clinical Bacteriology and Department of Biochemistry, Faculty of Medicine, Fatih University, Ankara, Turkey; ^2^Department of Infectious Diseases and Clinical Bacteriology, Fatih Sultan Mehmet Training and Research Hospital, 34746 Istanbul, Turkey; ^3^Department of Emergency Medicine, Faculty of Medicine, Yıldırım Beyazıt University, Ankara, Turkey; ^4^Department of Biochemistry, Faculty of Medicine, Yıldırım Beyazıt University, Ankara, Turkey; ^5^Department of Internal Medicine, Faculty of Medicine, Ankara University, Ankara, Turkey; ^6^Department of Biochemistry, Faculty of Medicine, Namik Kemal University, Tekirdağ, Turkey; ^7^Department of Oncology, Faculty of Medicine, Bahcesehir University, Istanbul, Turkey

## Abstract

*Background and Aim*. The aim of this study was to determine the actions of caffeic acid phenethyl ester (CAPE) on the changes of endothelin-1 (ET-1) level, tumor necrosis factor- (TNF-) alpha, and oxidative stress parameters such as superoxide dismutase (SOD) activities and malondialdehyde (MDA) levels in experimental sepsis model in rats. *Materials and Methods*. Twenty-four rats were randomly divided into three experimental groups: sham (group 1), sepsis (group 2), and sepsis + CAPE (group 3), *n* = 8 each. CAPE was administered (10 *µ*mol/kg) intraperitoneally to group 3 before sepsis induction. Serum ET-1, serum TNF-alpha, tissue SOD activity, and tissue MDA levels were measured in all groups. *Results*. Pretreatment with CAPE decreased ET-1, TNF-alpha, and MDA levels in sepsis induced rats. Additionally SOD activities were higher in rats pretreated with CAPE after sepsis induction. *Conclusion*. Our results demonstrate that CAPE may have a beneficial effect on ET and TNF-alpha levels and oxidative stress parameters induced by sepsis in experimental rat models. Therefore treatment with CAPE can be used to avoid devastating effects of sepsis.

## 1. Introduction

Sepsis is a complex process characterized by uncontrolled systemic inflammation that may eventually lead to multiorgan dysfunction and even death [1].

Endothelin-1 (ET-1) is the main isoform of the endothelin family, which have important roles in a wide range of diseases affecting the vascular system, kidney, heart, and lungs. Overexpression of ET-1 during sepsis, as a result of endothelial dysfunction, causes imbalance in local tissue perfusion [2, 3].

Excessive release of proinflammatory mediators which are induced by endotoxin like tumor necrosis factor-alpha (TNF-alpha) and reactive oxygen species (ROS) causes systemic inflammatory response during sepsis [4]. TNF-alpha has important roles in the development of organ dysfunction related to sepsis. It induces organ damage through activation of neutrophils and endothelial cells as well as coagulation abnormalities in patients with sepsis. Based on this knowledge, inhibition of TNF-*α* might be critical for treating septic organ dysfunction [5, 6].

ROS have been associated with the onset, progression, and outcome of sepsis, both in experimental and in clinical studies [7, 8]. Excessive production of ROS during sepsis is the result of actions of leukocytes and may disturb the antioxidative and oxidative balance resulting in organ damage leading to multiorgan failure [9]. Treatment options that may improve any of these parameters may be beneficial to treat sepsis.

Caffeic acid phenethyl ester (CAPE) is one of the major components of honeybee propolis and has been used in traditional medicine. It was found to be a potent free radical scavenger and antioxidant [10]. CAPE inhibits 5-lipooxygenase-catalysed oxygenation of linoleic acid and arachidonic acid in the micromolar concentration range [10]. It blocks production of reactive oxygen species (ROS) in human neutrophils and the xanthine/xanthine oxidase system at the concentration of 10 *μ*M [10]. It has been reported that CAPE is a potent anti-inflammatory and antioxidant agent and possesses cytostatic, antiviral, antibacterial, and antifungal properties [11].

The purpose of the current study was to evaluate the effects of CAPE on ET-1 levels, TNF-alpha, and oxidative stress in a rat sepsis model.

## 2. Materials and Methods

### 2.1. Study Design

Animal experiment was performed in accordance with the National Institute of Health guidelines for animal research and was approved by the Animal Research Ethics Committee of Fatih University School of Medicine, Istanbul. 24 adult male Wistar rats (weight range, 250 to 270 g) were used for the experiment. All animals had access to commercial standard diet and water ad libitum throughout the study. The rats were divided randomly into three groups of eight animals each: sham group (group 1), sepsis group (group 2), and sepsis group treated with CAPE (group 3).

Sham group (group 1) received only 1 mL intraperitoneal (i.p.) injection of 0.9% saline solution. Sepsis in group 2 and group 3 was induced by intraperitoneal (i.p.) injection of 2 × 10^10^ CFU of* Escherichia coli *ATCC 25922.* E. coli *ATCC 25922 was grown in brain-heart infusion broth. In the logarithmic phase of the growth, the suspension was centrifuged at 1000 g for 15 minutes, the supernatant was discarded, and the bacteria were resuspended and diluted in sterile saline. The rats received an i.p. inoculum of 1 mL of saline containing 2 × 10^10^ CFU of* E. coli *ATCC 25922 [12]. Six hours after bacterial challenge, group 2 received isotonic sodium chloride solution and group 3 received CAPE (10 *μ*mol/kg) by intraperitoneal injection. The CAPE was synthesized by standard method of Grunberger to prepare 25 micromol/mL of CAPE solution [13].

### 2.2. Sample Collection

24 hours after bacterial challenge, all animals were sacrificed using ketamine and cardiac puncture. Blood samples were drawn from vena cava inferior; liver tissue samples were immediately removed and stored at −80°C for the determination of tissue associated malondialdehyde (MDA) levels and superoxide dismutase (SOD) activities, as the parameters of oxidative stress. Blood samples were collected for the determination of endothelin and TNF-alpha levels, centrifuged at 3000 g for 10 minutes, and stored at −80°C.

### 2.3. Quantitative Determination of Serum Endothelin Levels

Rat big ET-1 levels were measured using commercially available enzyme-linked immunosorbent assay (ELISA) kits (Assay Designs, MI, USA) following the manufacturer's instructions. The results are presented as pg/mL.

### 2.4. Quantitative Determination of Serum TNF-Alpha Levels

Tumor necrosis factor-alpha rat ELISA kits produced by Biovendor Research and Diagnostic Products were utilised as described by the manufacturer. The results were expressed as pg/mL.

### 2.5. Quantitative Determination of Tissue Malondialdehyde Levels

Reaction with thiobarbituric acid (TBA) at 90–100°C was used as the basis for the determination of tissue thiobarbituric acid-reactive substance (TBARS) levels [14]. In the TBA test, a pink pigment with a maximum absorption at 532 nm was produced from the reaction of MDA or MDA-like substances with TBA. The reaction took place at pH 2-3 and 90°C for 15 min. Protein was precipitated by mixing samples with two volumes of cold 10% (w/v) trichloroacetic acid. The precipitate was pelleted by centrifugation and an aliquot of the supernatant was combined with an equal volume of 0.67% (w/v) TBA and placed in a boiling water-bath for 10 min. The absorbance was read at 532 nm after cooling. Results were expressed as nmol per gram wet tissue, using the standard graphic prepared based on measurements with a standard solution.

### 2.6. Quantitative Determination of Tissue Superoxide Dismutase Activities

SOD activity was determined according to the method defined by Sun et al. [15]. This method is based on the inhibition of nitroblue tetrazolium (NBT) reduction using a xanthine-xanthine oxidase system as a superoxide generator. Following the addition of 1.0 mL of an ethanol-chloroform mixture (5 : 3, v/v) to an equal volume of sample and centrifugation, activity was assessed in the ethanol phase of the supernatant. One unit of SOD was defined as the amount causing 50% inhibition in the NBT reduction rate. SOD activity is expressed as U mg^−1^ protein.

### 2.7. Statistical Analysis

For statistical evaluation, we used the statistical software package SPSS 15.0 and probability value of less than 0.05 was accepted as statistically significant. As the data were independent and showed normal distribution, statistical analysis was performed using analysis of variance (ANOVA) followed by Tukey's test when comparing groups. The results are given as the mean ± standard deviation of the mean (SD).

## 3. Results

**Figure 1:**
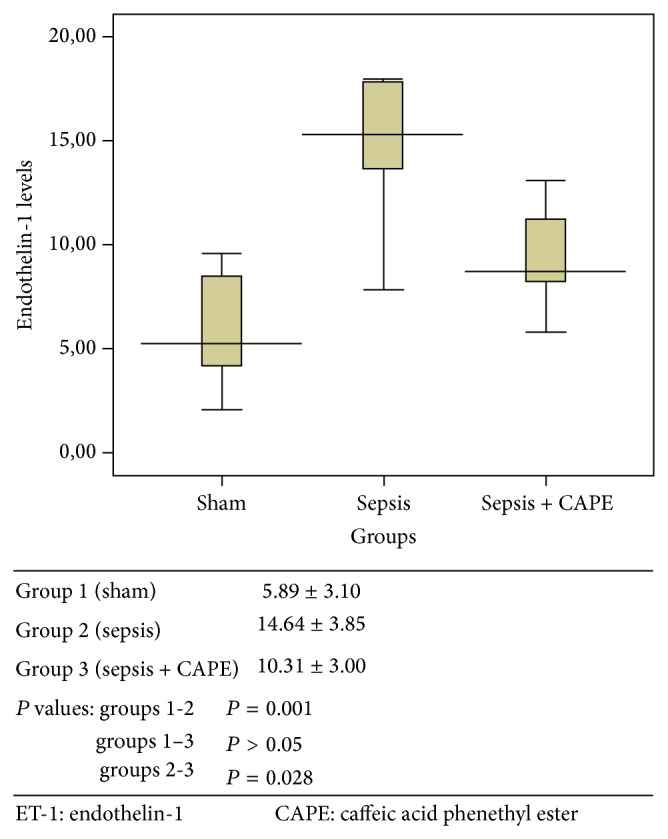
Serum ET-1 level (pg/mL) was described as mean value ± SD for all groups.

**Figure 2:**
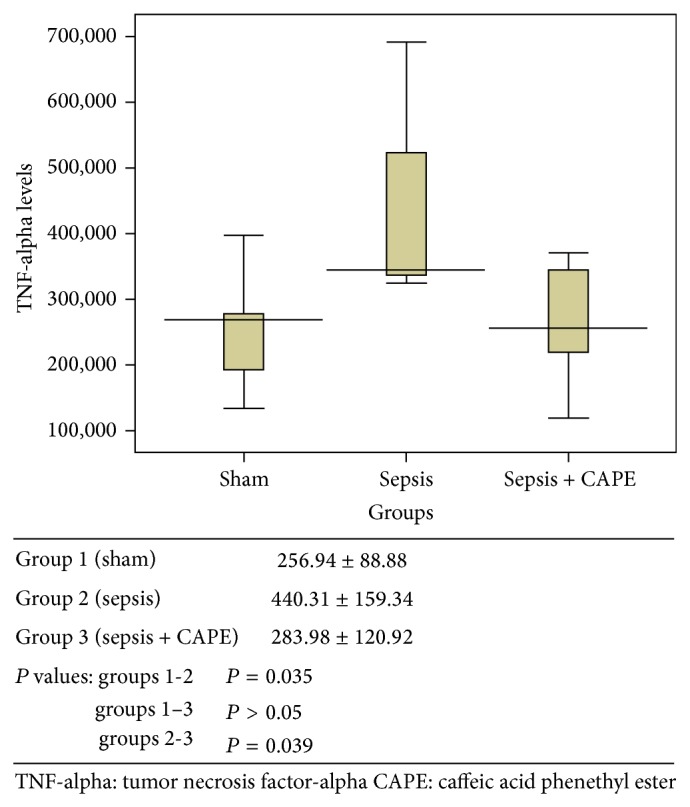
Serum TNF-alpha level (pg/mL) was described as mean value ± SD for all groups.

**Figure 3:**
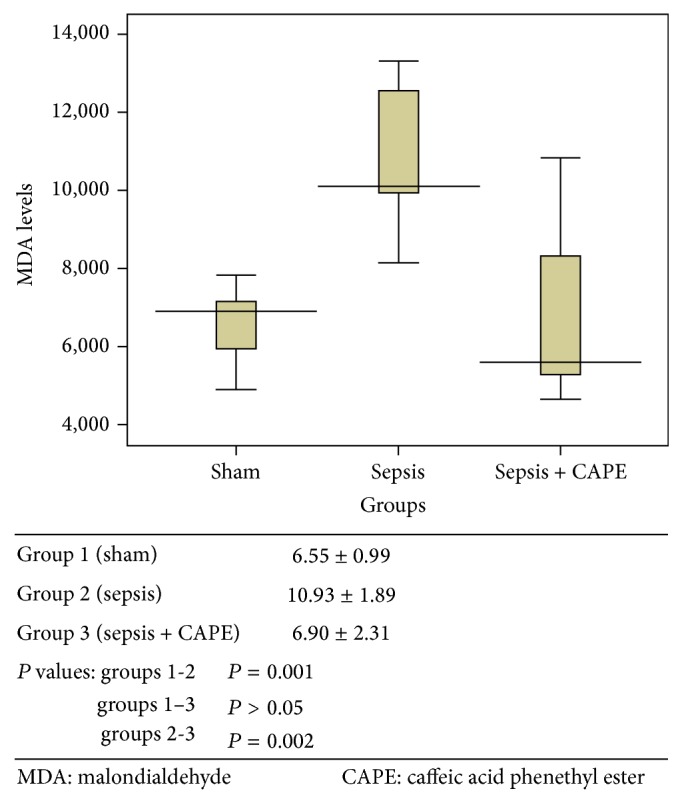
Tissue MDA level (nmol g^−1^ protein) was described as mean value ± SD for all groups.

**Figure 4:**
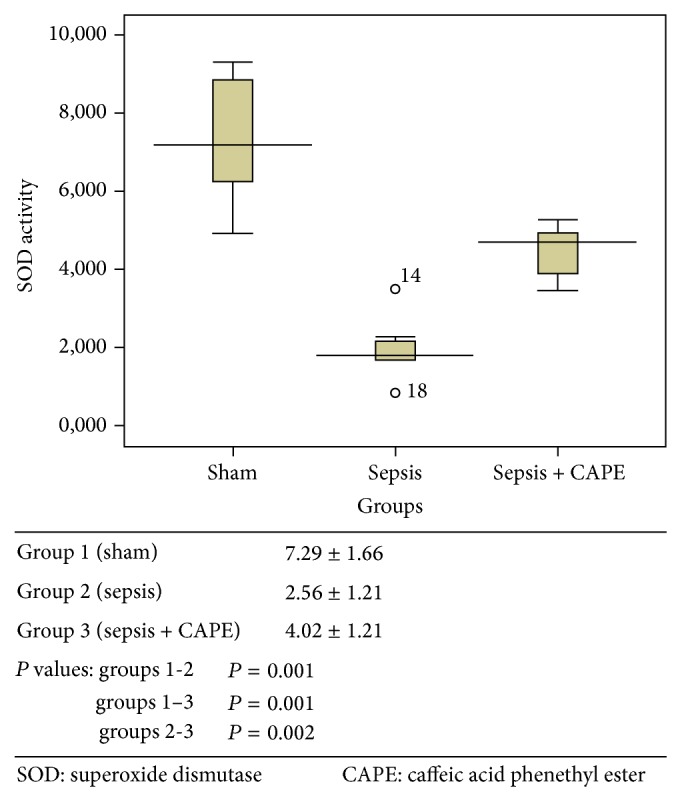
Tissue SOD activity (U mg^−1^ protein) was described as mean value ± SD for all groups.

ET-1, TNF-alpha, and MDA levels and SOD activities for all groups are presented in Figures 1–4 as mean ± SD.

### 3.1. Serum Endothelin Levels

ET-1 levels were determined as it is one of the major peptides in the pathogenesis of sepsis (Figure 1). ET-1 levels in sepsis group treated with CAPE (group 3) were lower than in sepsis group (group 2) (*P* = 0.028). Similarly, ET-1 levels in sham group (G1) were lower than in sepsis group (group 2) (*P* = 0.001).

### 3.2. Serum TNF-Alpha Levels

TNF-alpha levels were determined since they have an important role in the pathogenesis of sepsis (Figure 2). TNF-alpha levels in group 2 were significantly higher compared to group 1 (*P* = 0.035). When sepsis was induced in rat pretreated with CAPE (group 3), TNF-alpha levels were found to be similar to group 1 (*P* > 0.05) and significantly lower compared to group 2 (*P* = 0.039).

### 3.3. Tissue Malondialdehyde Levels

Tissue MDA levels, used as a marker of oxidative stress, were determined (Figure 3). Tissue MDA levels were significantly increased in the sepsis group (group 2) compared to group 1 (*P* = 0.001). When rats were pretreated with CAPE before sepsis induction, MDA levels were significantly lower compared to those in group 2 (*P* = 0.002).

### 3.4. Tissue Superoxide Dismutase Levels

SOD activities were used as a marker of oxidative stress (Figure 4). Tissue SOD activities after sepsis induction (group 2) were significantly decreased relative to those in group 1 (*P* = 0.001). When rats were pretreated with CAPE prior to sepsis induction, SOD activities were significantly higher compared to group 2 (*P* = 0.002). Tissue SOD activities in group 1 were higher than group 3 (*P* = 0.001).

## 4. Discussion

Overall, results revealed that pretreatment with CAPE significantly reduced serum ET-1, TNF-alpha, and MDA levels while increasing SOD activities, when sepsis was induced in a rat sepsis model.

Sepsis syndromes range from the systemic inflammatory response syndrome to severe sepsis and septic shock. These syndromes are the major causes of death in critical care units worldwide [16]. Although none performed it in a systematic way, some studies have correlated inflammatory markers, oxidative stress, and clinical markers of organ failure [17].

ET-1 levels increase with a septic process in correlation with the circulatory dysfunction and sepsis severity [2, 18]. Its levels are thought to correlate with circulatory dysfunction during sepsis and with sepsis severity [19]. Several ET-1 receptor antagonists have already been reported to have possible beneficial effects on cardiovascular performance and survival in experimental sepsis models [20, 21]. Our results reveal that pretreatment with CAPE may also have an effect to decrease ET-1 levels during sepsis.

Increased oxidative stress, one of many factors involved in development of multiorgan dysfunction syndrome during sepsis, is the result of imbalance between antioxidant and oxidant status. ROS levels rise due to increased production as a part of the proinflammatory response during sepsis [17, 22]. Moreover, during sepsis, major endogenous antioxidant systems including glutathione, vitamins A, C, and E, and several other antioxidant enzymes such as SOD are commonly depleted [22, 23]. SOD activity, an enzyme that scavenges the superoxide radicals and catalyzes them to hydrogen peroxide and oxygen, is commonly used to provide information on the antioxidant status [24]. MDA is also used to determine oxidant status, as it is a product of lipid peroxidation [25].

TNF-alpha is one of the proinflammatory cytokines which has been accepted as important mediators of sepsis [26–28]. Therefore, we wanted to investigate that therapeutic strategies may reduce the severity of sepsis by decreasing proinflammatory cytokines like TNF-alpha. In previous investigations, Pascual et al. described inhibition of the tumor necrosis factors pathway by propolis and Jung WK et al. reported decreasing serum levels of tumor necrosis factor-alpha and interleukin-1 beta by treatment of CAPE in lipopolysaccharide-induced septic shock model of mice [29, 30]. Our study results also have shown that TNF-alpha levels are lower upon pretreatment with CAPE. This finding also supports that CAPE may have beneficial effects during sepsis.

Death from sepsis has decreased recently depending on various adjunctive therapies. However, it remains high when compared with other critical illnesses [31]. In addition to fluid resuscitation, antibiotic therapy, and source control to remove the sepsis-inducing insult, inotropic-vasopressor therapy, glycemic control, prophylaxis for deep vein thrombosis, and stress ulcer prophylaxis are involved in sepsis treatment [16]. However, widespread research for a new drug that is effective in reducing mortality in sepsis has been sustaining.

A growing number of reports demonstrate that a proinflammatory and oxidative condition is related to the pathogenesis and the progression of endotoxin-induced septic shock and that antioxidants may have therapeutic potential in lipopolysaccharide- (LPS-) induced sepsis [32].

Because of this, in current study we investigate the roles of antioxidant therapy using CAPE in the treatment of sepsis. CAPE is a major component of honey bee propolis which is commonly used in Chinese traditional medicine. CAPE was also investigated in various organs such as intestine, lung, and spinal cord in a number of animal models and it has been reported that it has anti-inflammatory, radical scavenging and, therefore, antioxidant activities [10, 11, 33–37]. It also has immunomodulatory actions such as inhibition of ROS production and it is also demonstrated to exhibit cytostatic, antiviral, antibacterial, and antifungal properties [10, 11, 38–45]. Korish and Arafa showed that CAPE decreased the inflammatory cytokines and increased the anti-inflammatory cytokines levels in septic shock model and suggested that CAPE could help the prophylaxis and treatment of septic shock [46]. Similarly, Teke et al. reported that CAPE significantly decreased oxidative stress in another intraperitoneal sepsis model [47]. Moreover, there have been no reported side effects of CAPE on normal cells [45].

## 5. Conclusion

Our study demonstrated that effects of CAPE seemed to depend not only on the diminution of oxidative damage but also on its anti-inflammatory activity in rats in vivo. Therefore, it is reasonable to propose CAPE as a molecule with therapeutic potential for the treatment of systemic inflammation by interfering at the earliest steps of activation of the oxidative and proinflammatory cascade.

These data demonstrate the protective effect of CAPE treatment in experimental sepsis by reducing the inflammatory process and oxidative stress.
